# Surface Friction Gradient-Mediated Directional Liquid Transport under Vibrational Activation

**DOI:** 10.1021/acs.langmuir.5c03044

**Published:** 2025-09-02

**Authors:** Hong-Ren Jiang, Ming-kuan Wu, Jia-Cheng Song, Ting-Kuang Chung, Bing-Han Jiang

**Affiliations:** † Institute of Applied Mechanics, 33561 National Taiwan University, No.1, Sec. 4, Roosevelt Rd., Da’an Dist., Taipei City 106, Taiwan (R.O.C.)

## Abstract

This study introduces a method for directional liquid transport by leveraging surface friction gradients coupled with vibrational activation. We engineered friction gradients on polydimethylsiloxane (PDMS) substrates through controlled silicone oil infiltration, achieving significant variations in contact angle hysteresis. Surface characterization confirmed that higher silicone oil concentrations reduced hysteresis, enabling enhanced droplet mobility. Using a motorized vibrational platform, we systematically explored the interplay of vibration parameters, droplet size, and surface properties. High-speed imaging revealed that droplets exhibit asymmetric contact-line motion under optimized vibrational conditions, driving persistent migration toward higher-friction regions. The technique’s versatility was demonstrated through guided droplet transport and controlled coalescence on dual-gradient substrates, with applications validated for merging droplets. These findings offer a robust framework for designing droplet manipulation systems.

## Graphical abstract

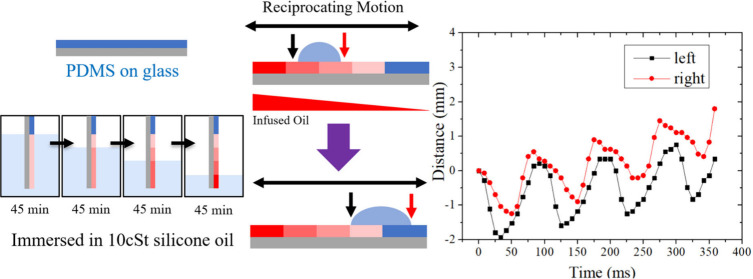

## Introduction

The controlled transport of liquid droplets on surfaces plays a crucial role in various technological applications,^1,2^ from water collection systems to liquid-handling devices.^3,4^ While droplet manipulation can be achieved through various active methods such as electrowetting,^5^ magnetic fields,^6,7^ or mechanical actuation,^8,9^ passive approaches utilizing surface property gradients offer promising alternatives due to their simplicity and versatility.^10^

The challenge lies in generating sufficient driving forces to overcome contact line pinning while maintaining reliable directional control over droplet movement.

Early investigations into gradient-driven droplet transport focused on chemical modification of surfaces to create wettability gradients, which demonstrated that water droplets could move spontaneously up inclined surfaces through carefully designed surface chemistry gradients.^11^ This concept was further developed to understand droplet motion on gradient surfaces.^12^ Building on these foundations, Researchers explored the dynamics of droplets on surfaces with varying chemical properties.^13,14^ However, surface energy gradients alone often provide limited driving forces, making it difficult to achieve efficient long-distance transport, particularly for larger droplets.

Recent years have seen growing interest in combining surface gradients with external activation to enhance transport capabilities. It has been demonstrated that vibration could significantly enhance droplet mobility on gradient surfaces^15^ and asymmetric vibration could drive droplet climbing against gravity.^9^ Furthermore, asymmetric surfaces designed using pulsed laser techniques can facilitate liquid transport through vibrational activation or boiling states,^16–18^ while engineered surface lubrication patterns enable spontaneous droplet self-propulsion.^19^ These studies revealed the potential of coupling surface properties with dynamic forcing to achieve controlled droplet movement, showing how periodic forcing can help overcome static pinning forces. These studies revealed the potential of coupling surface properties with dynamic forcing to achieve controlled droplet movement, showing how periodic forcing can help overcome static pinning forces.^8^

Recent studies have established a fundamental connection between surface friction and contact angle hysteresis in the context of droplet mobility.^20,21^ Surface friction in liquid–solid interfaces specifically refers to the resistance encountered by the three-phase contact line during droplet movement, which manifests macroscopically as contact angle hysteresis—the difference between advancing and receding contact angles (θ_A_ – θ_R_).^22^ This hysteresis directly quantifies the energy barrier that must be overcome for droplet motion to occur.^23^ Surfaces with high friction exhibit pronounced contact angle hysteresis, resulting in strong droplet pinning and reduced mobility. Conversely, low-friction surfaces demonstrate minimal hysteresis, allowing droplets to move with reduced resistance. The magnitude of this friction force per unit length of contact line can be expressed as *f*_friction_ = γ­(cos θ_R_ – cos θ_A_), where γ represents the liquid–air surface tension.^22^ This relationship has been extensively validated by experimental studies^24,25^ and provides a direct quantitative link between measurable contact angles and friction forces. By engineering spatial gradients in surface friction through controlled surface modification, it should become possible to create preferential pathways for droplet movement when coupled with appropriate activation mechanisms.

The emergence of liquid-infused surfaces has opened new possibilities for controlling surface friction and droplet mobility.^26–29^ Smith et al. demonstrated that lubricant-infused surfaces can dramatically reduce contact angle hysteresis.^26^ These advances suggest the possibility of creating well-defined friction gradients through controlled surface lubrication, potentially offering more robust control over droplet movement than traditional chemical gradients.

Building on these developments, we present a novel approach for achieving directional droplet transport through the integration of engineered surface friction gradients with vibrational activation. Our method utilizes controlled infiltration of silicone oil into PDMS^30^ polymer networks to create stable surfaces with spatially varying friction properties. Through precise control of the dip-coating process, we generate continuous gradients in surface friction, which, when combined with appropriate vibrational forcing, enable reliable directional transport of water droplets. This work demonstrates that millimeter-scale droplets can be effectively transported through the coupling of surface friction gradients with vibrational activation. The millimeter-scale droplets are particularly relevant for several emerging applications such as high-throughput biochemical assays^31^ and water harvesting systems.^32,33^ This study reveals how droplet size, vibration parameters, and surface properties interact to determine transport behavior. The results provide insights into contact line dynamics on gradient surfaces while establishing practical design principles for droplet manipulation systems.

## Experimental Method

### Materials and Surface Preparation

Glass substrates (75 × 25 mm, thickness 1.7 mm) were used as base materials. Polydimethylsiloxane (PDMS) was prepared by thoroughly mixing Sylgard 184 silicone elastomer base and curing agent at a weight ratio of 10:1. After degassing in a vacuum chamber for 30 min to remove air bubbles, the mixture was poured into a mold to achieve a uniform thickness of 1 mm. The PDMS-coated substrates were then cured at 70 °C for 2 h in a convection oven. The friction gradient was established using a self-made programmable dip-coating system with silicone oil (viscosity: 10 cSt) as the primary lubricant. The concentration gradient was achieved through controlled withdrawal speeds.

### Surface Characterization

Surface wettability was characterized through static and dynamic contact angle measurements. Contact angle hysteresis was determined by measuring the difference between advancing and receding angles during substrate tilting. Images of droplets are analyzed by ImageJ. Measurements were performed at 2 mm intervals along the gradient direction to map the wettability distribution.

### Vibration System and Motion Analysis

A computer-controlled motorized stage (maximum speed: 300 mm/s) provided vibrational actuation. The substrate mounting system incorporated custom-designed clamps to ensure stable positioning during experiments. Droplet motion was captured using high-speed imaging at 1000 frames per second, with the camera positioned perpendicular to the substrate surface. Image analysis was performed using ImageJ software to track droplet position and morphological parameters.

## Results and Discussion

To achieve controlled directional liquid transport on surfaces, we developed a systematic approach to create well-defined friction gradients through precise control of surface oil modification. The surface modification process involved creating a controlled variation in silicone oil concentration along the substrate (Figure 1(a)).

**Figure 1:**
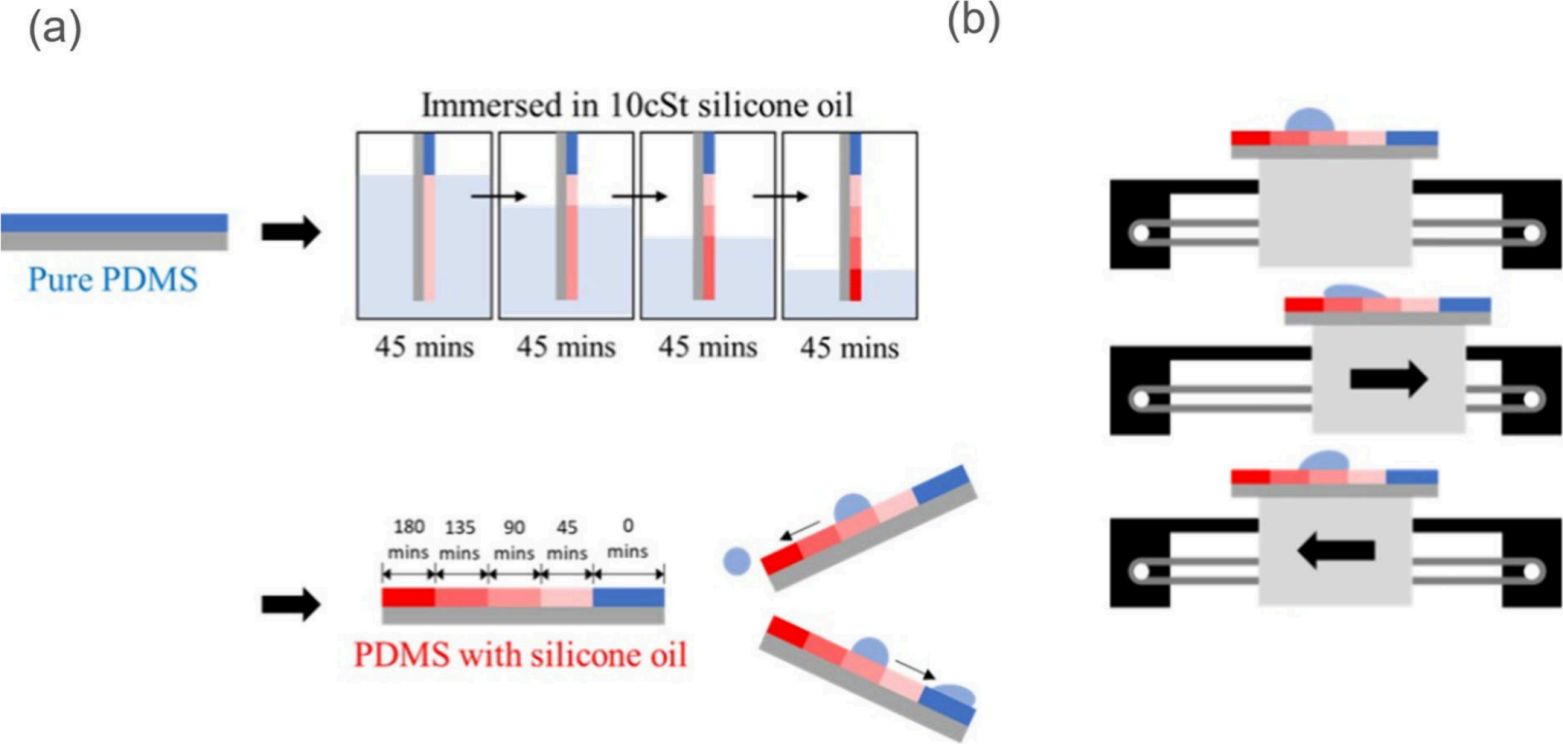
Experimental setup and surface modification process. (a) Schematic illustration of the surface friction gradient preparation method using silicone oil treatment. The process involves controlled dip-coating to establish a continuous gradient of surface friction along the substrate. Four distinct regions with different immersion times (45–180 min) create a well-defined friction gradient. (b) Experimental setup showing the motorized platform used for vibrational activation. The platform provides controlled oscillatory motion to induce directional droplet transport along the friction gradient.

The mechanism by which silicone oil infiltration creates a friction gradient can be understood through its interaction with the PDMS polymer network.^30,34^ PDMS contains a cross-linked polymer backbone with unbound oligomers. When exposed to silicone oil, the oil gradually infiltrates the polymer network, causing swelling and modification of the surface properties.^30^ Longer immersion times allow for greater oil penetration depth and more complete surface coverage, resulting in progressively reduced contact angle hysteresis.

We employed either a single-stage immersion process or a multistage approach to establish precise lubrication gradients. For the multistage process, we established four distinct regions with systematically increasing immersion times (45, 90, 135, and 180 min), which resulted in a stepped gradient of surface modification. Through experimental observation, we found that droplets exhibited preferential mobility characteristics: they readily slid toward regions exposed to longer silicone oil immersion times while showing increased adhesion in areas with shorter immersion periods, as demonstrated in Figure 1(a). This differential behavior confirms the successful creation of a functional friction gradient along the substrate surface.

The observed directional preference in droplet mobility directly correlates with the engineered friction gradient on the substrate surface. In this context, surface friction specifically refers to the resistance encountered by the contact line during droplet movement, which is quantitatively characterized by contact angle hysteresis. Our wettability analysis (Figure 2) confirms that regions with longer silicone oil immersion times exhibit significantly reduced contact angle hysteresis (from 55° to 8°), indicating lower surface friction. This reduction occurs because the infiltrated silicone oil creates a thin lubricating layer that minimizes direct interaction between the water droplet and the underlying PDMS substrate, effectively lowering the energy barrier for contact line movement. Consequently, the spatial variation in contact angle hysteresis translates to a well-defined friction gradient across the substrate, with the friction force decreasing gradually from the nonimmersed region toward areas subjected to longer immersion periods. This gradient in surface friction provides the fundamental driving mechanism for directional liquid transport under vibrational activation.

**Figure 2:**
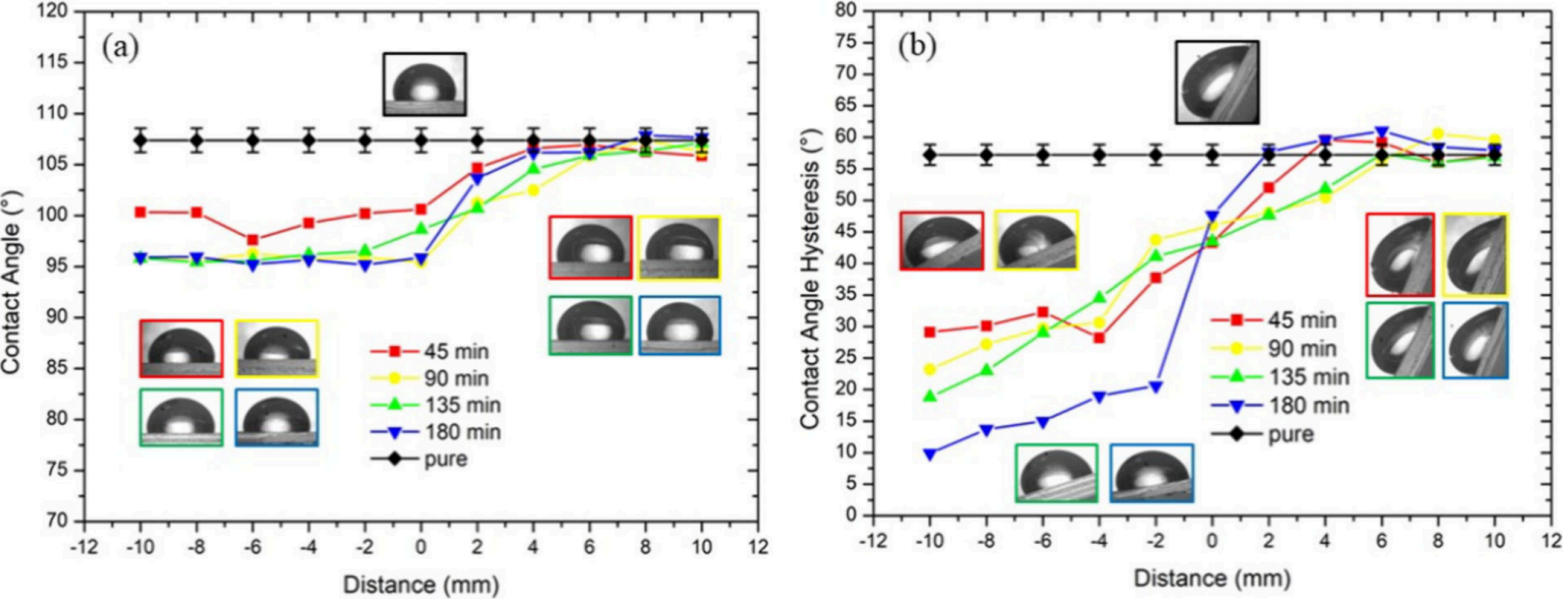
Contact angle hysteresis and sliding behavior analysis. (a) Contact angle hysteresis versus position along the gradient surface. (b) Sliding angle measurements at different gradient positions.

To systematically investigate the transport behavior, we designed and implemented a computer-controlled motorized platform capable of providing precise vibrational actuation (Figure 1(b)). Our initial experimental focus centered on elucidating the relationship between platform oscillation speed and droplet transport efficiency. In these experiments, we utilized standardized 50 μL water droplets while maintaining a constant vibration amplitude of 2.5 mm, systematically varying the platform speed across a range of 20 to 250 mm/s. (also see Movie S1). The results, presented in Figure 3(a), revealed distinct threshold behavior in the transport dynamics. At lower speeds (below 50 mm/s), droplets exhibited minimal movement. However, once the critical threshold was exceeded, droplets demonstrated consistent directional transport, with transport velocity showing a positive correlation with platform speed. A particularly noteworthy observation was the emergence of a characteristic dual-phase motion pattern, consisting of an initial stationary period followed by directed movement. This behavior was consistently observed throughout our experiments, except at the highest speeds where immediate directional motion occurred without the initial stationary phase.

**Figure 3:**
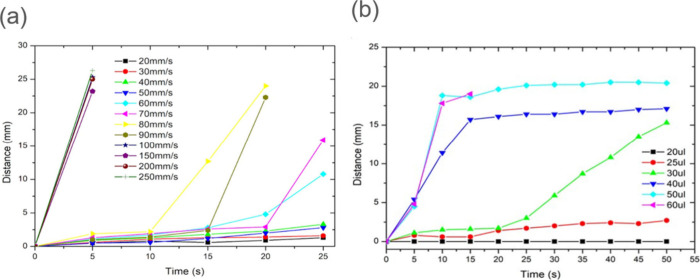
Droplet transport dynamics under vibrational activation. (a) Analysis of droplet transport velocity as a function of platform oscillation speed. The plot demonstrates the existence of a critical speed threshold (approximately 50 mm/s) below which no directional transport occurs, followed by increasing transport efficiency at higher speeds. (b) Size-dependent transport behavior showing the relationship between droplet volume and directional movement. Results indicate a critical volume threshold around 30 μL, below which directional transport becomes ineffective.

We further investigated the influence of droplet size on transport efficiency (Figure 3(b)). Under fixed conditions (platform speed: 200 mm/s, amplitude: 2.5 mm), we observed a critical volume threshold around 30 μL. Smaller droplets (20–25 μL) showed no directional transport, while larger droplets exhibited consistent directed motion. Droplets near the critical volume (30 μL) demonstrated interesting transitional behavior, typically remaining stationary for an initial period before initiating sustained directional movement.

To understand the underlying transport mechanism, we conducted detailed analysis of droplet deformation and contact line dynamics. Imaging revealed that moving droplets underwent significant elongation during transport (Figure 4(a)). Quantitative analysis of droplet length versus position demonstrated a strong correlation between droplet deformation and transport velocity, with periods of rapid movement corresponding to increased elongation (Figure 4(b)).

**Figure 4:**
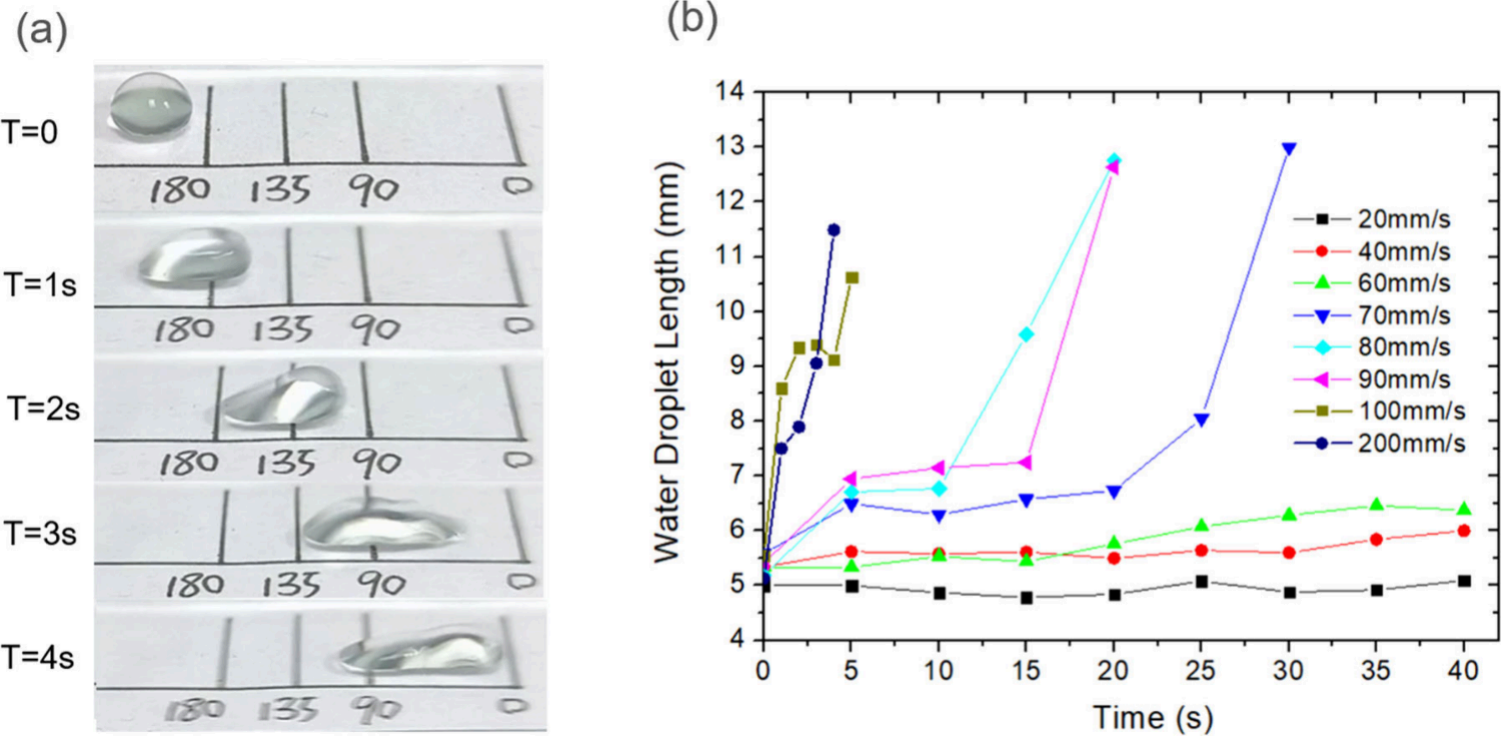
Droplet deformation analysis during transport. (a) Time-sequence images showing the evolution of droplet shape during vibrational activation. The images capture the characteristic elongation of the droplet during transport. (b) Quantitative analysis of droplet length variation with position under different vibrational speeds, demonstrating the correlation between droplet deformation and transport efficiency.

High-speed imaging analysis of contact line dynamics (Figure 5) revealed fundamentally distinct behavioral patterns that were strongly dependent on droplet volume. Our investigation demonstrated that smaller droplets (30 μL) manifested limited asymmetric motion in their contact lines (Figure 5(a)), which resulted in negligible net displacement across the surface. In marked contrast, larger droplets (50 and 70 μL) exhibited pronounced asymmetric motion patterns as shown in Movie S2 for 50 μL droplets and Figure 5(b) for 70 μL droplets, facilitating substantially more efficient directional transport. This volume-dependent behavior was quantitatively characterized through position–time analysis (Figures 5(c) and 5(d)), which showed that larger droplets achieved equivalent spatial displacement with approximately an order of magnitude greater efficiency, completing the same translation distance in one-tenth of the time required by smaller droplets.

**Figure 5:**
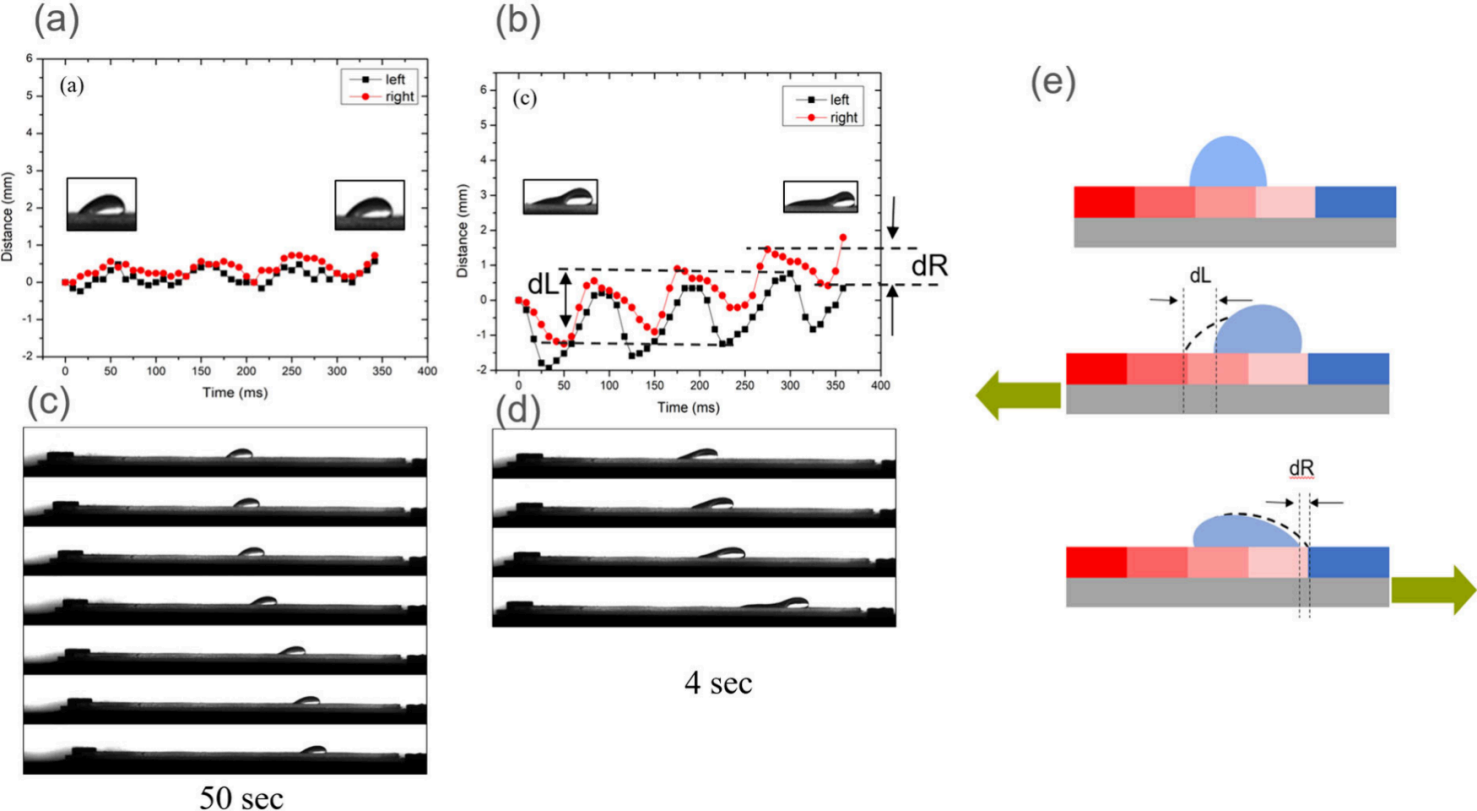
Detailed contact line dynamics and transport mechanism. (a) Analysis of contact line trajectories for a 30 μL droplet, showing limited asymmetric motion and minimal net displacement. (b) Contact line trajectories for a 70 μL droplet, demonstrating enhanced asymmetric motion and more effective directional transport. (c) Position–time plot for a 30 μL droplet over 50 s, illustrating relatively slow transport dynamics. (d) Position–time plot for a 70 μL droplet over 4 s, showing significantly faster directional transport. (e) Schematic illustration of the transport mechanism, where dL and dR represent the displacement of left and right contact lines during one vibration cycle. The asymmetric contact line motion leads to net directional movement.

The underlying transport mechanism was elucidated through detailed analysis of the asymmetric response of contact lines during individual vibration cycles (Figure 5(e)). The friction force per unit length of contact line can be expressed as

$$f_{\mathrm{friction}}=\gamma \left(\cos\;\theta_{R}-\cos\;\theta_{A}\right)$$ 1

where γ is the liquid–air surface tension, and θ_R_ and θ_A_ are the receding and advancing contact angles, respectively. For a droplet with contact radius *r*, the total friction force is approximately *F*_friction_ ≈ 2π*r*·*f*_friction_. On our gradient surface, the contact angle hysteresis Δθ = θ_A_ – θ_R_ increases along the *x*-direction (toward higher friction), creating a spatial gradient d*F*_friction_/d*x* > 0. When the substrate undergoes vibration with displacement *s*(*t*) = *A*sin­(ω*t*), the resulting inertial force acting on the droplet is

$$F_{\mathrm{vib}}\left(t\right)=-mA\omega^{2}\sin\left(\omega t\right)$$ 2

where *m* is the droplet mass, *A* is vibration amplitude, and ω is angular frequency. Our mechanism requires two simultaneous conditions to achieve directional transport:

$$\left|F_{\mathrm{vib}}\right|>F_{\mathrm{friction}}\left(x\right)$$ 3a

$$F_{\mathrm{friction}}\left(x+\Delta x\right)>F_{\mathrm{friction}}\left(x\right)$$ 3b

The first condition ([Disp-formula eq3a]) ensures that the vibration force exceeds the initial friction threshold, allowing contact-line depinning and droplet motion during the positive half-cycle (toward higher friction). The second condition ([Disp-formula eq3b]) represents the essential friction gradient, ensuring that after initial displacement Δ*x*, the droplet encounters stronger pinning at its new position.

During the positive half-cycle, when condition ([Disp-formula eq3a]) is satisfied, the droplet displaces forward by δ_+_ ≈ (*F*_vib_ – *F*_friction_(*x*))/*k*_cap_, where *k*_cap_ is an effective capillary stiffness. In the negative half-cycle, the reversed *F*_vib_ attempts recovery, but the higher friction leads to incomplete retraction δ_–_ < δ_+_, due to asymmetric pinning. This yields net displacement per cycle Δ*x* ≈ δ_+_ – δ_–_ ∝ (d*F*_friction_/d*x*)

This dual-condition mechanism explains why both sufficient vibration force and a friction gradient are necessary for directional transport. Without adequate vibration force, no initial motion occurs. Without a friction gradient, the motion would be symmetric, resulting in zero net displacement. The observed size dependence arises because larger droplets experience greater inertial forces relative to surface friction, enabling them to more effectively overcome the initial pinning threshold. As droplet volume increases, the radius *r* increases as V^1/3^, leading to a proportional increase in the absolute pinning force. However, the increased droplet mass simultaneously increases the vibration-induced force *F*_vib_.

We analyzed the force balance in our system. With our platform operating at 200 mm/s with 2.5 mm amplitude (nominal frequency ∼ 10 Hz), the nonharmonic motion during acceleration/deceleration phases likely enhances the effective acceleration experienced by droplets, and we take 50% increase in acceleration compared to pure harmonic motion. We estimated the force ratio between vibration-induced force, *F*_vib_ and surface friction *F*_friction_ with a contact angle hysteresis of 25°. For a 30 μL droplet, *F*_vib_ is 4.4 × 10^–4^ N and *F*_friction_ is 3.8 × 10^–4^ N, which explains why such volumes exhibit threshold behavior. For larger droplets (50–70 μL), ratios of *F*_vib_/*F*_friction_ are 1.6–2, consistent with their reliable transport observed experimentally. This analysis supports the proposed mechanism and aligns with the experimental observation that droplets exceeding 30 μL demonstrate consistent directional motion on our friction gradient surfaces.

To validate the practical utility of this transport mechanism, we engineered specialized substrates incorporating dual friction gradients, which established converging transport pathways (Figure 6). This innovative design architecture enabled precise control over droplet coalescence at predetermined locations along the substrate surface. Through careful gradient engineering, we achieved controlled droplet convergence at a central meeting point, specifically within the nonimmersion region of the substrate. The temporal evolution of this coalescence process is comprehensively documented in Figure 6(a), which illustrates the synchronized movement and subsequent merging of two separate droplets. The underlying mechanism of this controlled coalescence is schematically represented in Figure 6(b), demonstrating how the opposing friction gradients effectively guide individual droplets toward a designated convergence point, ultimately facilitating their controlled merger. Our experiments involve water–water merging where liquid properties (surface tension, viscosity) remain unchanged during the coalescence process. Compared to other liquid-infused surfaces, our method utilizes a relatively small amount of infiltrant oil with immersion times of tens of minutes, which does not reach the equilibrium saturation time of several hours typically required for PDMS-silicone oil systems.^30^ As a result, there is no distinct silicone oil layer on the surface; instead, the silicone oil remains embedded within the PDMS matrix, thereby minimizing interference with droplet properties.

**Figure 6:**
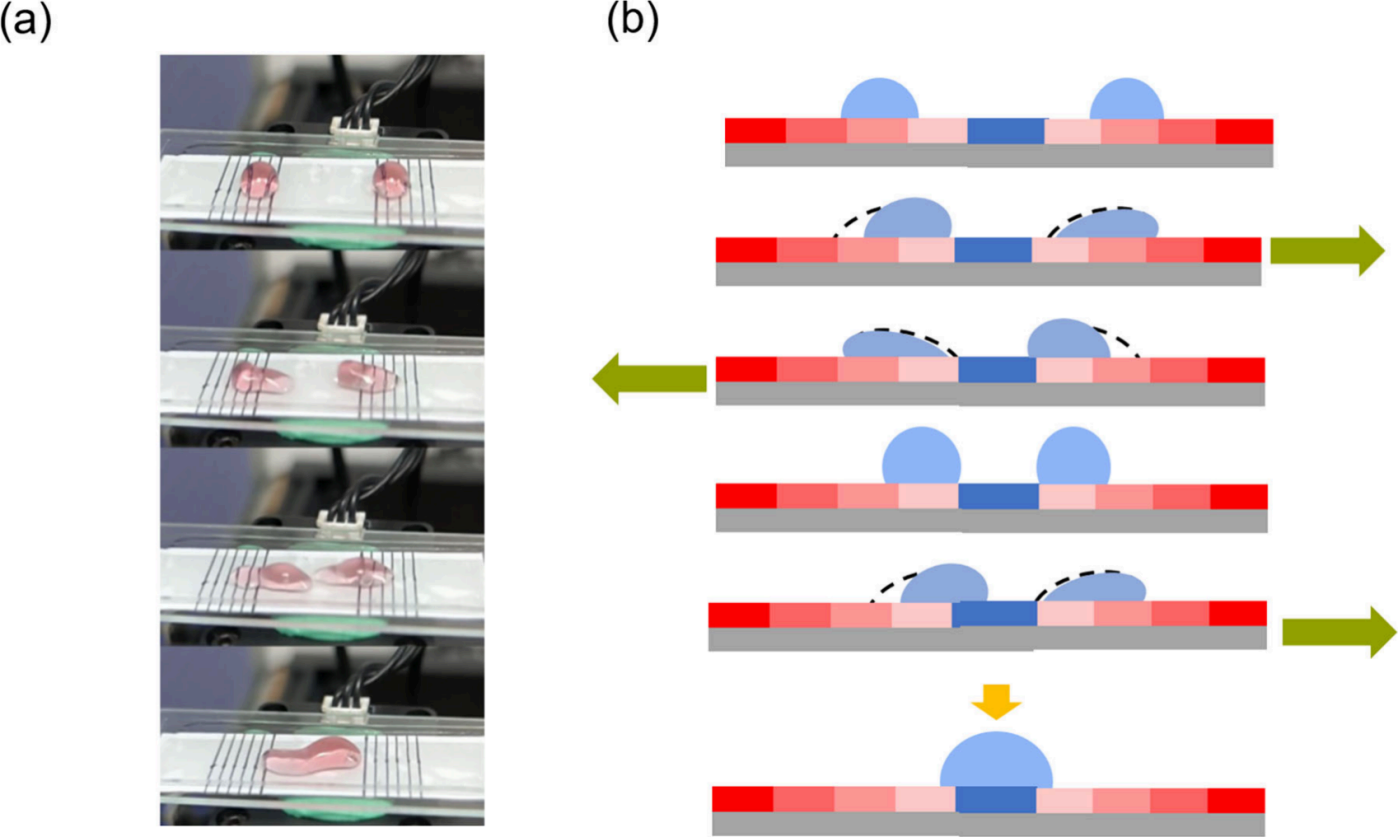
Controlled droplet coalescence application. (a) Time-lapse sequence showing controlled merging of two droplets under vibrational activation. The process demonstrates precise spatial control of droplet movement and coalescence. (b) Schematic representation of the dual-gradient design principle, illustrating how opposing friction gradients guide droplets toward a central meeting point for controlled coalescence.

Unlike electrowetting or magnetic actuation approaches, our friction gradient-based method offers key advantages: it operates with ordinary water droplets without specialized components, enables passive directional transport through built-in surface properties, and can be fabricated using simple, low-cost methods. These features make it promising for resource-limited applications and integrated microfluidic systems requiring robust liquid handling capabilities. The vibrational requirements are modest, requiring approximately 1–2 mm amplitude and 10 Hz frequency, which can be practically achieved using inexpensive vibration motors for cost-effective and energy-efficient applications. Our current infiltration method can be further enhanced through masking techniques. Due to the slow diffusion rate of silicone oil in thin samples, which limits lateral spreading, different masks can be employed to selectively expose specific regions to oil treatment, enabling the creation of complex gradient patterns such as curved or circular motion paths for advanced droplet manipulation applications. Our experimental findings demonstrate that droplets move toward regions of higher surface friction upon vibrational activation. For liquid collection applications, this principle can be exploited by designing masks that leave designated collection areas untreated (without silicone oil infiltration), thereby creating convergence zones for efficient droplet harvesting.

## Conclusions

We have demonstrated a novel approach for droplet transport by exploiting the asymmetric contact line dynamics created by surface friction gradients under vibrational activation. The key mechanism relies on the differential pinning forces experienced by advancing and receding contact lines across the gradient, leading to directional transport when coupled with appropriate vibrational forcing. This understanding enabled practical applications including controlled droplet coalescence through dual-gradient designs. The physical framework developed here provides a foundation for designing surfaces with prescribed friction gradients for precise droplet manipulation in microfluidic applications.

## Supplementary Material





## Data Availability

The data sets used and/or analyzed during the current study available from the corresponding author on reasonable request.

## Notes

Movie S1, a 50 μL water droplet exhibiting spontaneous directional motion from low-hysteresis region (longer silicone oil immersion time) toward high-hysteresis region (shorter immersion time) under vibrational activation (the substrate was subjected to sinusoidal vibration with velocity of 200 mm/s and amplitude of 2.5 mm, and the droplet demonstrates characteristic asymmetric deformation during transport, with enhanced mobility at the leading edge (low-hysteresis region) and increased pinning at the trailing edge (high-hysteresis region)) (MP4)
Movie S2, high-speed side-view imaging of 50 μL droplet transport on surface friction gradient, played at 1/10 speed (identical conditions to Movie S1, showing detailed droplet deformation and contact line dynamics during directional transport) (AVI)
